# Targeting lactate transport suppresses *in vivo* breast tumour growth

**DOI:** 10.18632/oncotarget.3910

**Published:** 2015-05-14

**Authors:** Filipa Morais-Santos, Sara Granja, Vera Miranda-Gonçalves, António H.J. Moreira, Sandro Queirós, João L. Vilaça, Fernando C. Schmitt, Adhemar Longatto-Filho, Joana Paredes, Fátima Baltazar, Céline Pinheiro

**Affiliations:** ^1^ Life and Health Sciences Research Institute (ICVS), School of Health Sciences, University of Minho, Campus of Gualtar, Braga, Portugal; ^2^ ICVS/3B's - PT Government Associate Laboratory, Braga/Guimarães, Portugal; ^3^ DIGARC - Technology School, Polytechnic Institute of Cávado and Ave, Barcelos, Portugal; ^4^ IPATIMUP - Institute of Molecular Pathology and Immunology of University of Porto, Porto, Portugal; ^5^ Medical Faculty of the University of Porto, Porto, Portugal; ^6^ Department of Pathology and Medicine, Laboratoire National de Sante, Dudelange, Luxembourg; ^7^ Molecular Oncology Research Center, Barretos Cancer Hospital, Barretos, Sao Paulo, Brazil; ^8^ Laboratory of Medical Investigation (LIM-14), School of Medicine, University of Sao Paulo, Sao Paulo, Brazil; ^9^ Barretos School of Health Sciences, Dr. Paulo Prata - FACISB, Barretos, Sao Paulo, Brazil

**Keywords:** monocarboxylate transporters, breast carcinoma, hypoxia, lactate, Warburg effect

## Abstract

**Background:**

Most cancers, including breast cancer, have high rates of glucose consumption, associated with lactate production, a process referred as “Warburg effect”. Acidification of the tumour microenvironment by lactate extrusion, performed by lactate transporters (MCTs), is associated with higher cell proliferation, migration, invasion, angiogenesis and increased cell survival. Previously, we have described MCT1 up-regulation in breast carcinoma samples and demonstrated the importance of *in vitro* MCT inhibition. In this study, we performed siRNA knockdown of MCT1 and MCT4 in basal-like breast cancer cells in both normoxia and hypoxia conditions to validate the potential of lactate transport inhibition in breast cancer treatment.

**Results:**

The effect of MCT knockdown was evaluated on lactate efflux, proliferation, cell biomass, migration and invasion and induction of tumour xenografts in nude mice. MCT knockdown led to a decrease in *in vitro* tumour cell aggressiveness, with decreased lactate transport, cell proliferation, migration and invasion and, importantly, to an inhibition of *in vivo* tumour formation and growth.

**Conclusions:**

This work supports MCTs as promising targets in cancer therapy, demonstrates the contribution of MCTs to cancer cell aggressiveness and, more importantly, shows, for the first time, the disruption of *in vivo* breast tumour growth by targeting lactate transport.

## BACKGROUND

Breast cancer is the most common cancer among women and the second leading cause of cancer-related mortality [1]. It is characterized by its clinical and molecular heterogeneity, as it is no longer seen as a single disease but rather as a multifaceted disease comprising distinct biological subtypes: luminal (A and B), HER2/neu+, normal breast-like, and basal-like [2, 3]. These molecular subtypes have important prognostic implications and different predictive values, being the basal-like subtype the most aggressive and with poorer prognosis, with no targeted therapy so far [4, 5]. Treatment of this breast cancer subtype with conventional chemotherapeutic agents maintains the risk of breast cancer recurrence substantially high, around 30–40% [6], reinforcing the urgent need to look for new therapeutic targets.

It is known that most solid tumours, including breast cancer, have high rates of glucose consumption associated with lactate production, even in the presence of sufficient oxygen to sustain oxidative phosphorylation, by a process known as “Warburg effect” [7, 8]. The high glycolytic rates can provide several advantages to cancer cells, namely the acidification of tumour microenvironment by lactate extrusion, which is associated with higher cell proliferation, migration and invasion, angiogenesis and increased cell survival [9, 10]. To maintain the glycolytic flux, cancer cells up-regulate several proteins, including glycolytic enzymes and pH regulators, such as monocarboxylate transporters (MCTs) that will mediate the efflux of lactate [11].

The MCT family, Solute Carrier Family 16 (SLC16), comprises fourteen related proteins, being MCT1 and MCT4 responsible for the efflux of lactate coupled with a proton across the plasma membrane [12-15], contributing to the acidic tumour environment, which is adverse to non-tumour cells. These transporters require a protein chaperone, CD147, to be trafficked to plasma membrane and perform their activity [16].

The enhanced rates of glycolysis and glucose uptake in tumours are maintained by several adaptive mechanisms, including adaptation to hypoxia conditions [17-19], in which HIF-1α (Hypoxia inducible factor 1-α) is a key player by regulating several metabolism related proteins like the glucose transporter 1 (GLUT1), carbonic anhydrase 9 (CAIX) and MCTs [17, 20, 21]. In breast cancer, our group reported an association between MCT1 and both GLUT1 and CAIX expression, particularly in the basal-like subtype [22], which is associated with shorter disease-free survival. Also, MCT1 and CD147, alone or in co-expression, were also associated with estrogen receptor (ER) and progesterone receptor (PR) absence, high histological grade and proliferative index (Ki67), and presence of basal markers such as cytokeratin 5, 14 and vimentin [23], supporting the role of MCT1/CD147 in breast cancer aggressiveness as well as in the maintenance of the glycolytic phenotype. Consistently, several authors have demonstrated the importance of MCT inhibition in cancer, using both *in vitro* and *in vivo* models [for review see [24]]. Further, Morais-Santos *et al.* characterized the effect of targeting MCTs, especially MCT1, in a panel of breast cancer cell lines. Activity inhibition of MCTs with different inhibitors (quercetin, lonidamine and α-cyano-4-hydroxycinnamic acid (CHC)) decreased *in vitro* breast cancer cell aggressiveness, decreasing glucose consumption and lactate production, cell viability, proliferation, migration and invasion. Also, in Hs578T cells, which express MCT1 but lower MCT4, impairment of lactate transport led to increased cell death by apoptosis. Specific inhibition of MCT1 by siRNA in the same cells corroborated the results obtained by activity inhibition [25]. Also, results from the group in lung cancer and in glioblastoma models also anticipate the success of targeting MCTs [26, 27].

Thus, the promising results that have been emerging in the last years point at MCTs as new promising anticancer targets, however, more studies are needed to validate the potential of lactate transport inhibition in breast cancer treatment. In this study, we show that MCT1/4 knockdown reduced lactate transport, cell aggressiveness *in vitro* and, more importantly, inhibited tumour formation and reduced *in vivo* tumour growth.

## RESULTS

### Cancer cell metabolism is remodelled by hypoxia

A panel of three basal-like breast cancer cell lines, with different levels of MCT1/4 expression [25], was specifically used in this study to assess the importance of each MCT isoform in tumour progression.

**Figure 1:**
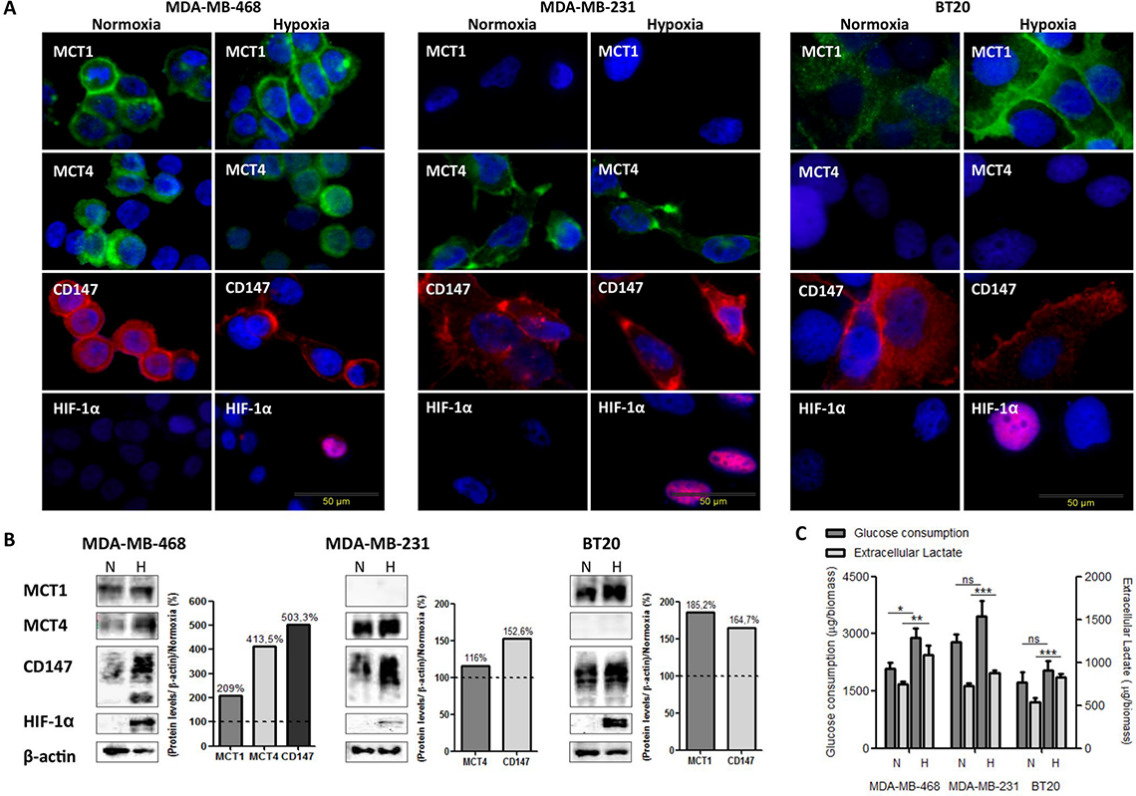
The metabolic profile of breast cancer cell lines is modulated by hypoxia **A.** Immunofluorescence staining of breast cancer cell lines for MCT1 (green), MCT4 (green), CD147 (red) and HIF-1α (red) after 24 hours under normoxic (N) and hypoxic (H) conditions (magnification, x400; DAPI- blue nuclear staining). **B.** Western blot analysis of MCT1 (50kDa), MCT4 (52kDa), CD147 (31–65 kDa) protein expression under normoxic (N) or hypoxic (H) conditions. β-actin was used as loading control. The graphs represent protein quantification of each blot, compared with normoxia (dashed line). **C.** Glucose consumption and extracellular lactate production by breast cancer cell lines after 24 hours of normoxic (N) or hypoxic (H) conditions. Results are the mean of at least three independent experiments in triplicate ± SEM. *: *p* < 0.05; **: *p* < 0.01; ***: *p* < 0.001 normoxia *vs*. hypoxia.

MCT1, MCT4 and CD147 expression under normoxia and hypoxia conditions is shown in Figure 1A. In MDA-MB-468 cells, MCT1 and CD147 were found in the cytoplasm and plasma membrane, while MCT4 was found in the cytoplasm, being maintained in hypoxia (Figure 1A). MDA-MB-231 cells which do not express MCT1 [28], showed MCT4 and CD147 expression at both the cytoplasm and plasma membrane in normoxic and hypoxic conditions (Figure 1A). In BT20 cells, the reinforcement of MCT1 and CD147 plasma membrane expression after hypoxia is more evident, while MCT4, even after hypoxia, was not detected in this cell line (Figure 1A).

By Western blot analysis (Figure 1B), there was differential expression of MCTs and CD147 under hypoxia. In MDA-MD-468 cells, MCT1, MCT4 and CD147 protein expressions increased approximately 2-, 4- and 5-fold, respectively. Protein expression alteration in MDA-MB-231 cells was not so obvious, with only a 1.1- and 1.5-fold change in MCT4 and CD147, respectively. Finally, BT20 cells presented an increase in MCT1 of about 1.8-fold and an increase of CD147 of about 1.6-fold.

The glycolytic metabolism of the human breast cancer cell lines under hypoxia was evaluated by glucose and lactate quantification in the culture medium. All cell lines significantly increased the efflux of lactate, being accompanied by a significant increase in glucose uptake only in MDA-MB-468 cells (Figure 1C).

### Blocking lactate transport decreases the metabolic requirements to support cell aggressiveness

**Figure 2:**
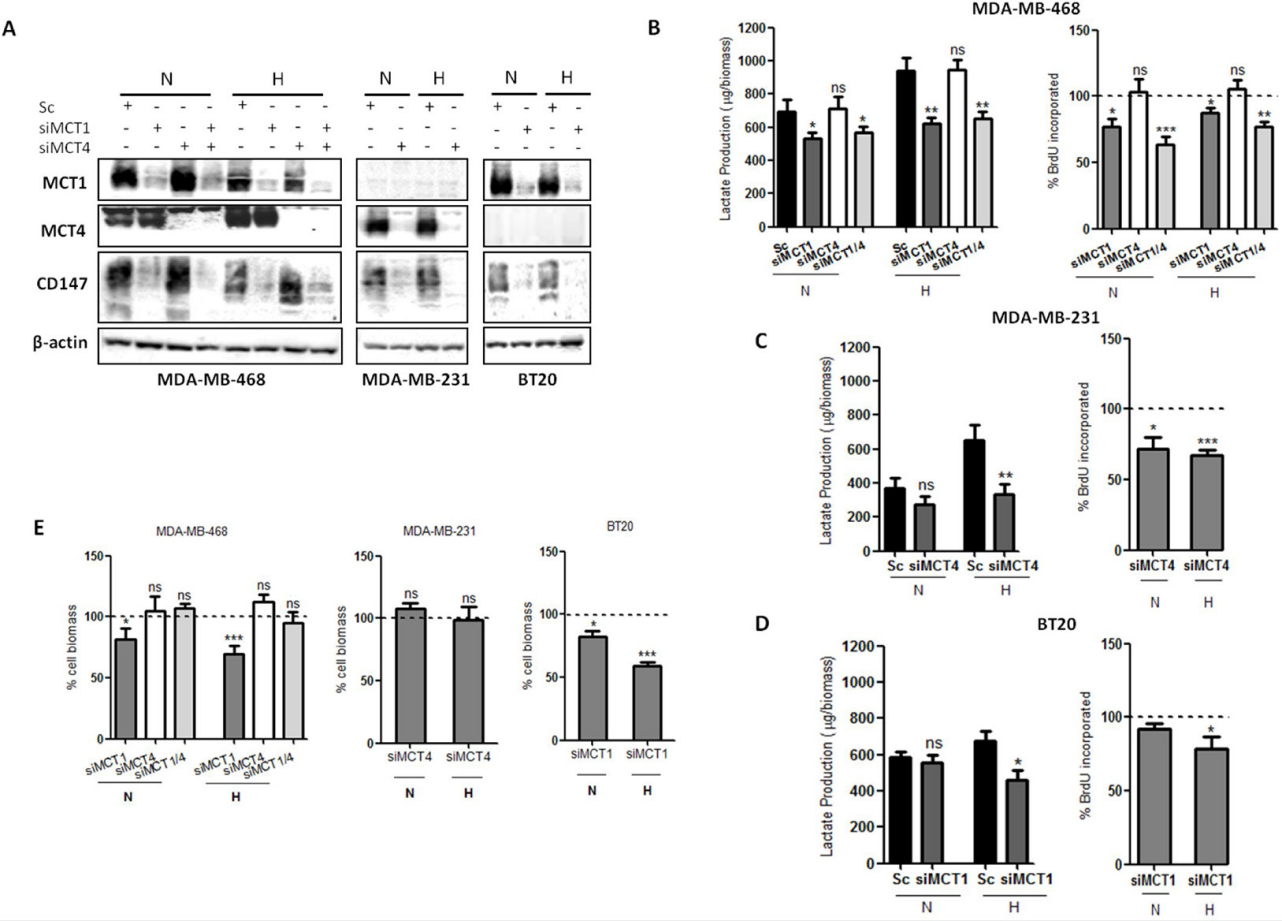
MCT knockdown impairs glycolytic metabolism and cell proliferation **A.** Western blot results for MCT1, MCT4 and CD147 in silenced breast cancer cell lines under 24 hours of normoxia (N) or hypoxia (H). β-actin was used as loading control. **B,C,D.** Lactate production by silenced cells in normoxia (N) and hypoxia (H) compared to control (Sc - scramble) and percentage of cell proliferation by BrdU incorporation of silenced cells comparing to control (dashed line) in normoxia (N) and hypoxia (H). **E.** Percentage of cell biomass of silenced cells compared to respective control (dashed line). Results are the mean of at least three independent experiments in triplicate ± SEM. *: *p* < 0.05; **: *p* < 0.01; ***: *p* < 0.001.

To better understand the role of each MCT isoform, a single transient knockdown for MCT1 (siMCT1), MCT4 (siMCT4) or double transient knockdown for MCT1 plus MCT4 (siMCT1/4) was performed, under normoxia and hypoxia conditions. As seen by Western blot (Figure 2A), MCT1 and MCT4 knockdown was almost complete in the three cell lines. Additionally, MCT knockdown was even more efficient in hypoxia (Supplementary Figure S1A). In MDA-MB-231 and BT20 cell lines, which only express one MCT isoform, MCT4 and MCT1 knockdown, respectively, was able to efficiently decrease the expression levels of CD147 (Figure 2A, Supplementary Figure S1A), while, in MDA-MB-468 cell line, only MCT1 or MCT1/4 knockdown was capable to reduce CD147 expression (Figure 2A and Supplementary Figure S1A).

In addition, in MDA-MB-468 cells, lactate release and cell proliferation were significantly decreased after MCT1 and double knockdown, in both normoxia and hypoxia, while MCT4 knockdown showed no effect (Figure 2B). Regarding MDA-MB-231 cells, MCT4 knockdown significantly reduced lactate secretion only in hypoxia conditions, while cell proliferation was significantly decreased under both normoxia and hypoxia (Figure 2C). In contrast, in BT20 cells, after MCT1 knockdown, lactate secretion and cell proliferation were only significantly decreased under hypoxic conditions (Figure 2D). The influence of MCT knockdown on cell biomass was also accessed and the results showed that only MCT1 knockdown induced a significant decrease on cell biomass, having a more profound effect under hypoxic conditions for both MDA-MB-468 and BT20 cells lines (around 30 and 40%, respectively) (Figure 2E).

**Figure 3:**
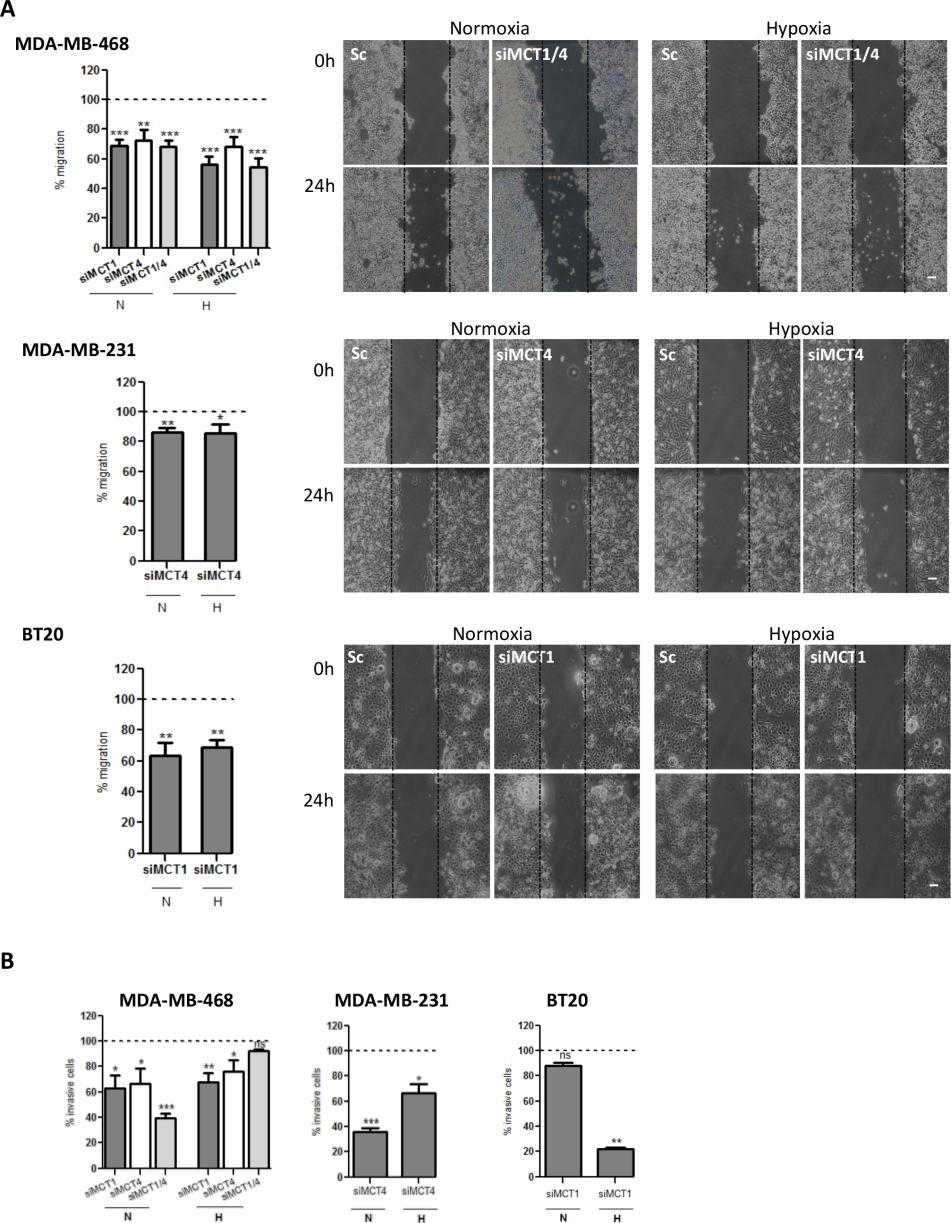
MCT knockdown decreases *in vitro* cell migration and invasion **A.** Percentage of cell migration after 24 hours of normoxia (N) or hypoxia (H). Silenced cells were compared with the respective control (dashed line). Representative pictures of cell migration at 0 hours and 24 hours are shown (scale bar 100 μm). **B.** Percentage of invasive cells after 24 h of normoxia (N) or hypoxia (H). Silenced cells were compared with the respective control (Sc - scramble). Representative pictures of cell invasion at 24 hours of normoxia (left panel) or hypoxia (right panel) are shown. Results are the mean of at least three independent experiments in triplicate ± SEM. *: *p* < 0.05; **: *p* < 0.01; ***: *p* < 0.001.

To evaluate if the decrease of lactate-induced acidification of the extracellular media influenced other tumourigenic features, we also analysed cell migration and invasion. Thus, as observed in Figure 3A, the single knockdown of MCT1 or MCT4 and the double knockdown were able to significantly decrease cell migration in all cell lines (Figure 3A and Supplementary Figure S1B), in both normoxia and hypoxia. Regarding cell invasion, in MDA-MB-468 cells, the percentage of invasive cells was significantly reduced in normoxia upon MCT1, MCT4 and MCT1/4 knockdown, while, in hypoxia, the inhibition of invasion caused by MCT knockdown was lost in siMCT1/4 (Figure 3B and Supplementary Figure S1C). MCT4 knockdown in MDA-MB-231 cells decreased cell invasion more effectively in normoxia, whereas MCT1 knockdown in BT20 cells only decreased significantly cell invasion in hypoxia (Figure 3B and Supplementary Figure S1C).

### MCT1/4 knockdown impairs tumour growth *in vivo*

**Figure 4:**
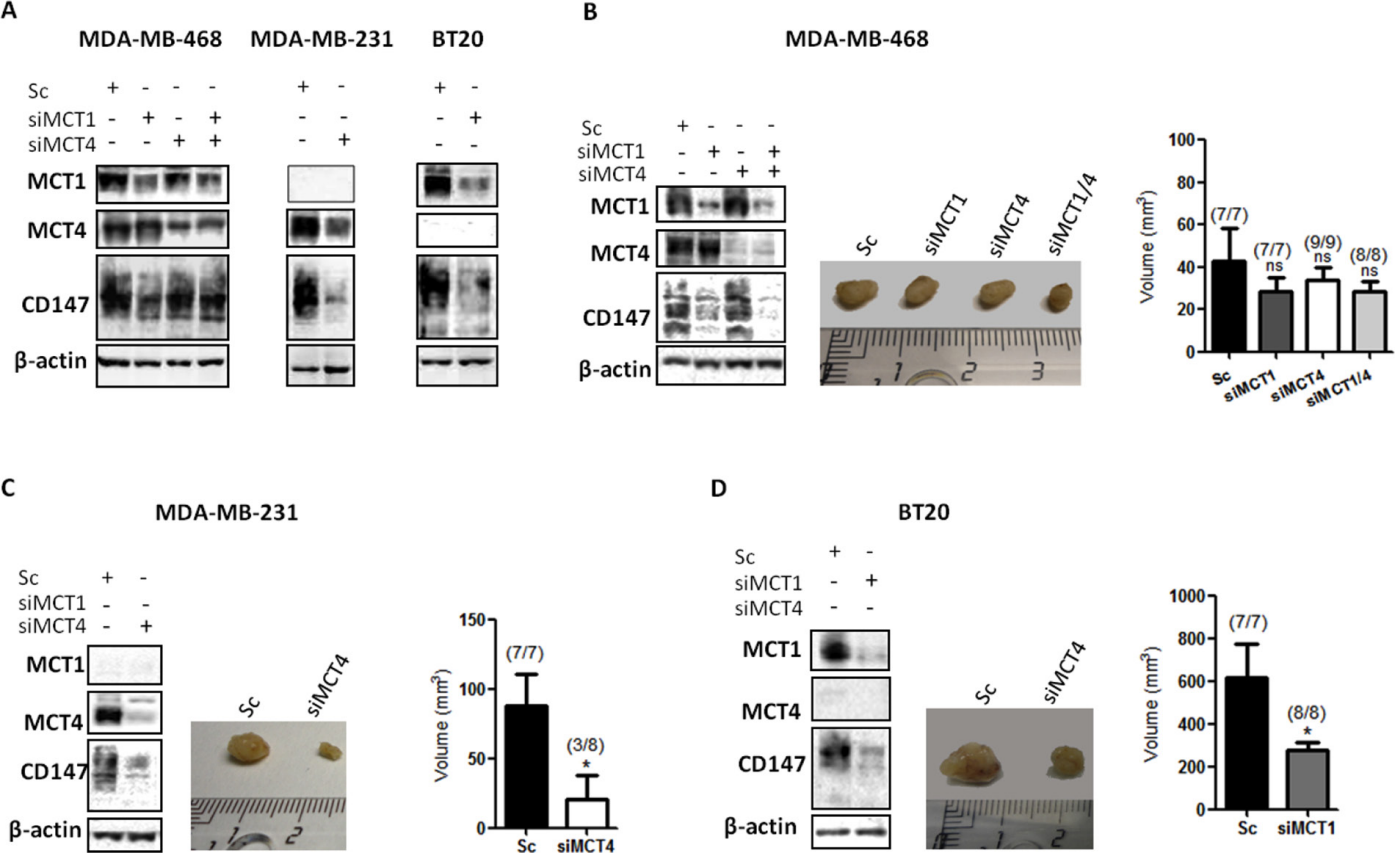
Knockdown of MCT1 and MCT4 decreases tumour volume *in vivo* **A.** Western Blot results showing MCTs knockdown after 10 days in MDA-MB-468 cells and 14 days in MDA-MB-231 and BT20 cells. β-actin was used as loading control. **B,C,D.** MCT1, MCT4 and CD147 protein expression evaluated by Western Blot at the time of injection in the mammary fat pad of nude mice of the different cell lines. Representative picture of excised tumours after 45 days (middle panel) and the respective tumour volume (mm^3^) in each animal group. The numbers in brackets indicate: number of animals with formed tumours/total number of animals in each group. The *in vivo* experiments were repeated twice. *: *p* < 0.05 siMCTs groups compared to scramble groups.

To test the *in vivo* role of MCTs during cancer initiation, MDA-MB-468, MDA-MB-231 and BT20 cells with transient MCT knockdown were injected into the mammary fat pad of nude mice and tumour formation and growth was monitored. The duration of *in vitro* silencing in cultured cells in normoxic conditions showed that, in MDA-MB-468 cells, silencing of MCT1 and MCT4 was stable up to 10 days (Figure 4A, Supplementary Figure S2A). However, CD147 depletion previously observed after MCT1 and MCT1/4 silencing (Figure 2A) was not maintained after 10 days of silencing (Figure 4A and Supplementary Figure S2A). On the other hand, in MDA-MB-231 and BT20 cells, MCT4 and MCT1 knockdown remained at least until 14 days, as well as CD147 (Figure 4A). The efficiency of MCT4 knockdown in MDA-MB-231 cells and MCT1 knockdown in BT20 cells is shown in Supplementary Figure S2A. Additionally, along 10 days of silencing, there are significant differences in cell proliferation for siMDA-MB-231 and siBT20 cells, and no difference for siMDA-MB-468 cells, compared to controls (Supplementary Figure S2B). The silencing levels at the time of injection are shown by Western blot in each cell line panel (Figure 4B, 4C and 4D).

In the *in vivo* model, MCT1 and MCT4 depletion resulted in a remarkable reduction of tumour growth for MDA-MB-231 and BT20 cells (Figure 4C and 4D). In particular, in MDA-MB-231 cells, at day 45 after injection, tumour volumes were 88 ± 23.4 mm^3^ in the control group *versus* 20 ± 17.8 mm^3^ in the MCT4 knockdown group. More importantly, in the MCT4 knockdown group, only 3 out of 8 injected animals developed tumours (Figure 4C). In BT20 cells, the depletion of MCT1 was also capable to significantly decrease tumour volume from 616 ± 162.4 mm^3^ in the control group to 278.8 ± 37 mm^3^ in the MCT1 knockdown group (Figure 4D). In this case, all the injected animals developed tumours. Finally, in MDA-MB-468 cells, the single or double MCT knockdown were not able to significantly reduce tumour volume or inhibit its formation (Figure 4B).

**Figure 5:**
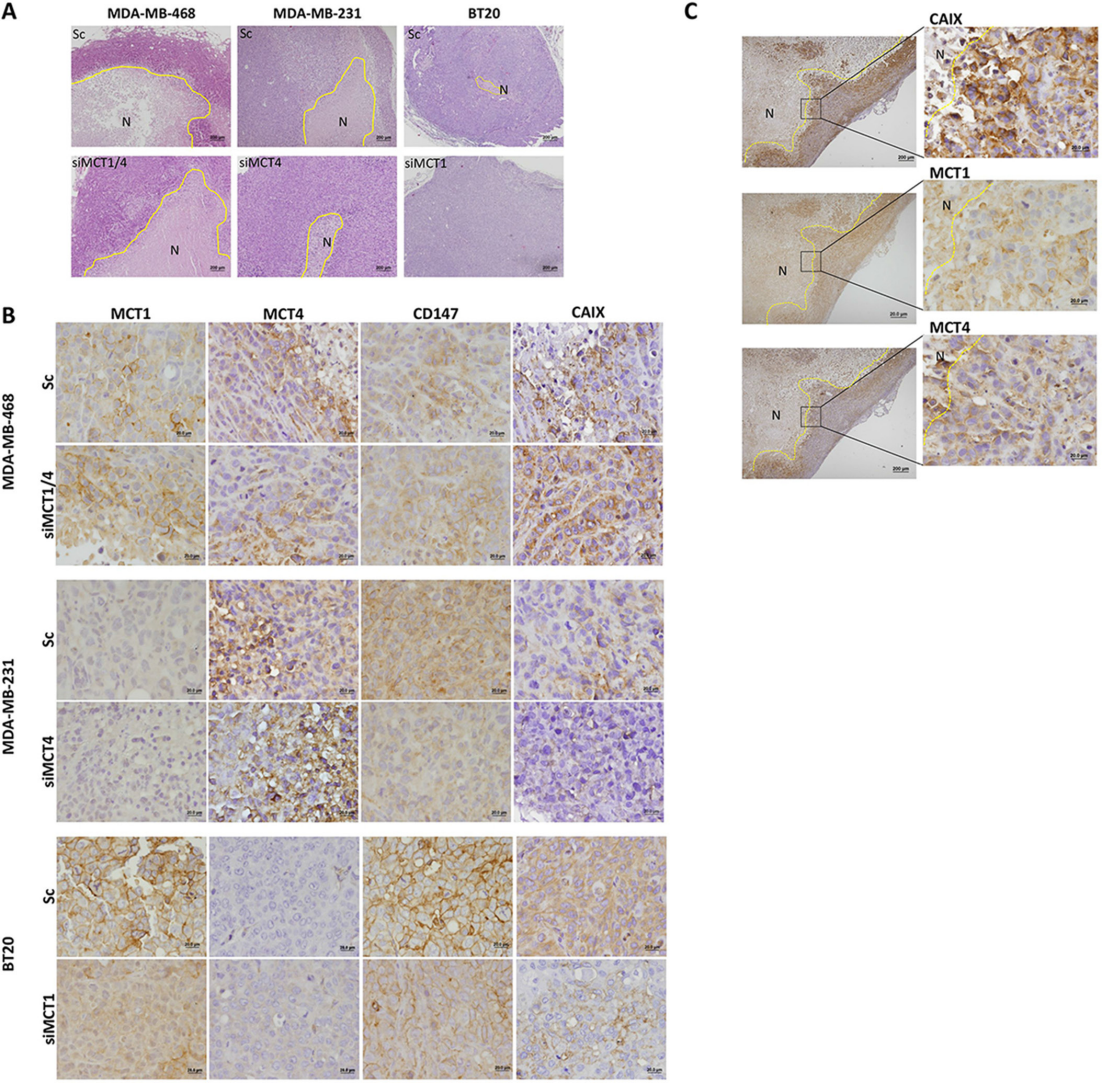
MCT knockdown inhibits metabolism-related protein re-expression at the plasma membrane **A.** Hematoxylin and Eosin staining of representative tumours. **B.** Immunohistochemical expression of MCT1, MCT4, CD147 and CAIX in the excised tumours. **C.** CAIX, MCT1 and MCT4 expression in siMCT1/MCT4 MDA-MB-468 tumours in peri-necrotic areas. The areas surrounded by the yellow line represent necrosis (N).

To investigate the effect of MCT knockdown in *in vivo* tumour growth inhibition, tumours were collected at the end of the experiment (day 45) and examined by immunohistochemistry for the expression of MCT1, MCT4, CD147 and CAIX (Figure 5B, 5C), CD31 and Ki67 (Supplementary Figure S2D). H&E staining demonstrated that MDA-MB-468 silenced and control groups presented high levels of necrosis, when comparing to MDA-MB-231 or BT20 tumours (Figure 5A). Also, after 45 days, MCTs were re-expressed in cancer cells, however, in MDA-MB-468 tumours, the expression of MCT1 and CD147 was predominantly at the plasma membrane in siMCT1, siMCT4 (Supplementary Figure S2C) and siMCT1/4 groups (Figure 5B), while for MDA-MB-231 and BT20 tumours, MCT1, MCT4 and CD147 re-expression was limited to the cytoplasm (Figure 5B). MDA-MB-231 and BT20 tumours did not express MCT1 and MCT4, respectively (Figure 5B). Results also showed that the levels of proliferation (Ki67) and vessel density (CD31) were not altered between control and knockdown groups (Supplementary Figure S2D). Finally, CAIX expression, a cancer cell pH regulator, was more evident in MDA-MB-468 scramble and knockdown tumour groups (Figure 5B) than in MDA-MD-231 and BT20 tumours, which expression was in its majority in the control groups (Figure 5B) compared to the silenced groups.

## DISCUSSION

Glycolytic metabolism has recently been proposed as a fundamental mechanism in the metabolic reprogramming of cancer cells [10]. In fact, the large amounts of glucose consumed by tumour cells has been useful in the diagnosis of breast cancer using ^18^FDG PET-scanning, particularly in the detection of metastases of primary tumours, recurrent disease and to monitor therapy response [29, 30]. As a consequence, several proteins are differentially expressed to sustain the glycolytic phenotype of tumour cells, like some pH regulators such as CAIX, MCTs and other proteins of the glucose pathway [17, 23]. Being basal-like tumours a very aggressive group of breast tumours [3, 5], without specific molecular therapy [4] and with high MCT1 expression, and very low positivity in the normal breast tissue [23], this molecule is seen as a promising therapeutic target for this breast cancer subtype.

In this work, we exposed breast cancer cell lines to hypoxia to enhance glycolysis and impair oxidative phosphorylation, a common feature in tumours with disrupted vasculature [8, 31]. Our results show a shift from oxidative phosphorylation to a more glycolytic phenotype in hypoxia, by increased lactate secretion, particularly in MDA-MB-468 and BT20 cells, as well as upregulation of the studied proteins, allowing cells to grow under intermittent hypoxia [9, 19] reinforcing the importance of glycolysis. Consequently, it is expected that hypoxic cells would be more dependent on MCT1/4 expression to export lactate, avoiding acid-induced necrosis and/or apoptosis. Thus, inhibition of MCTs by siRNA would be more effective in hypoxic conditions once cancer cells rely exclusively on glycolysis.

Our results show that MCT1 knockdown was more effective in hypoxia, with a higher decrease in lactate levels, cell biomass and cell invasion in BT20 cells. The drastic reduction observed in *in vivo* tumour growth corroborates these findings. Another study showed that treatment with metformin, which impairs oxidative phosphorylation forcing glycolysis, increase the response and the efficacy of MCT1 inhibitors [32]. Also, treatment of glycolytic cells, without MCT4, with a specific inhibitor of MCT1 showed a decrease in intracellular pH resulting in suppression of tumour growth [33]. Results from our group also demonstrated that *in vitro* silencing of MCT1 decreased lactate efflux, migration and invasion in both glioma and breast cancer cells [25, 27], as it happened with MCT activity inhibition using classical MCTs inhibitors [25].

Furthermore, silencing of MCT4 in MDA-MB-231 cells, in which MCT1 is silenced by methylation [28], was also able to decrease lactate secretion and proliferation, but not cell biomass. However, hypoxic conditions seem to have no influence *in vitro* comparing to normoxia, probably because MDA-MB-231 cells present a more pronounced glycolytic phenotype, with high glycolytic rates even in the presence of oxygen (Warburg effect). As expected, migration and invasion were also affected, probably due to the interaction between MCT4 and β1-integrin at the leading edge of migrating cells, as reported by others in the same breast cancer cell line [34]. Also, the high reduction of tumour volume demonstrates the great potential of targeting MCT4 in tumours where MCT1 is absent. This was also corroborated by the observed inhibition tumour initiation in 5 of 8 animals, upon depletion of MCT4. Additionally, ectopic expression of MCT4 in transformed fibroblasts (poor tumourigenic cells) completely restored tumourigenicity, pointing at MCT4 as a pro-tumoural molecule [33].

The combined silencing of MCT1 and MCT4 in MDA-MB-468 cells was expected to also have some effect in tumour reduction since, *in vitro*, we demonstrated a decrease in lactate secretion. However, it fails to reduce cell biomass and invasion in hypoxia. Moreover, MCT1 knockdown had also no effect in reduction of tumour volume *in vivo*. As MCT4 expression was mostly cytoplasmic, the results obtained for MCT4 knockdown in these cells were expected. As shown in previous work [25], this cell line presents lower MCT1 and MCT4 expression than the other two cell lines, which probably means a lower dependence on MCTs than MDA-MB-231 and BT20 cells, not showing tumour growth inhibition.

Interestingly, contrary to MDA-MB-231 and BT20 cells, after 10 days of silencing, MDA-MB-468 cells re-expressed CD147. This protein play an essential role in MCT trafficking to the plasma membrane and activity of MCT1 and MCT4, but also in the regulation of matrix metalloproteinases production [35]. Although other authors attributed the major pro-tumoural role of CD147 by chaperoning MCTs, other CD147 pro-tumoural roles, like interaction with signalling integrins, CD98/LAT1 complex and promotion of metalloproteinases were also proposed [33]. Once CD147 was re-expressed within a few days after MCT knockdown, this protein may induce proteins with a pro-tumoural function, like metalloproteinases, failing in the reduction of tumour volume even with low MCT expression, however more studies are needed to support this hypothesis. A particular finding was the high levels of necrotic areas in MDA-MB-468 tumours compared to the other tumour xenografts. Although several studies have reported necrosis as an expected result from MCT inhibition [36-38] the marked levels of necrosis in the control group, excludes MCT knockdown as the cause for tumour necrosis. The proliferation curves along 10 days of silencing show that MDA-MB-468 silenced cells proliferate at the same rate as control cells, supporting the *in vivo* results for this cell line. Moreover, as re-expression of MCT1 and CD147 was at the plasma membrane in MDA-MB-468 tumours, this will probably prevent the decrease of tumour growth. In contrast, in tumours in which MCT expression is restricted to one MCT isoform (MDA-MB-231 and BT20 tumours), re-expression of MCTs and CD147 after 45 days was only cytoplasmic, suggesting a possible disruption in the trafficking to the plasma membrane.

CAIX plays a role in the maintenance of intracellular pH levels of glycolytic cancer cells [39]. This protein was previously associated with MCT1 in a subset of breast cancer basal-like tumours and was also correlated with a shorter disease-free survival [22], pointing at CAIX as a marker of tumour aggressiveness. Also, in another study using invasive breast carcinomas, it was shown that overexpression of CAIX was correlated with poor prognosis [40]. Our present results showed CAIX expression in all control groups, probably as an initial response to tumour hypoxia, with a particular strong expression in MDA-MB-468 tumours and, in this case, being also expressed in the knockdown groups, probably contributing to the aggressive behaviour of tumour. There was a clear CAIX staining in the peri-necrotic areas with correspondence to either MCT1 or MCT4 positivity, supporting the role of CAIX in the maintenance of intracellular pH in glycolytic cells expressing MCT1/4. In contrast, in tumours which volume was reduced after MCT knockdown, re-expression of CAIX was almost insignificant.

Importantly, the increasing interest in metabolic-related targets, like MCT1, is driving the development of new classes of specific and high-affinity inhibitors, including the MCT1 specific inhibitors developed by AstraZeneca. Currently, a Phase 1 clinical trial (NCT01791595) is recruiting patients with prostate cancer, gastric cancer or diffuse large B cell lymphoma, to evaluate the maximum dose, the potential side effects of the drug and the pharmacokinetic profile of AZD3965, a specific inhibitor for MCT1/MCT2, demonstrating the pharmacological interest in targeting MCTs in cancer therapy [41].

## MATERIALS AND METHODS

### Cell culture

The human breast cancer cell lines MDA-MB-468, MDA-MB-231 and BT20 were obtained from ATCC or from collections developed at Drs Elena Moisseva (Cancer Biomarkers and Prevention Group, Departments of Biochemistry and Cancer Studies, University of Leicester, UK), Marc Mareel (Laboratory of Experimental Cancerology, Ghent University Hospital, Belgium) and Eric Lam (Imperial College School of Medicine, Hammersmith Hospital, London, UK).

All cell lines were routinely cultured in DMEM containing D-glucose (4,5 g/l), (Invitrogen), supplemented with 10% FBS (Invitrogen) and 1% penicillin–streptomycin (Invitrogen), in a 37°C humidified atmosphere with 5% CO_2_. For experiments cells were cultivated in DMEM without FBS. Hypoxia was achieved using a modular incubator chamber (MIC-101 Billups-Rothenberg Inc.), with an atmosphere of 95% nitrogen and 5% CO_2_, for 16 hours before starting the experiment, and maintained under hypoxia until the end of the experiment. Oxygen levels were monitored using an oxygen sensor (PAC 3500, Dräger) and never exceeded 1% at the end of the experiment.

### RNA interference and transfection

Silencing experiments were performed using 5 nM of Silencer Select Validated siRNAs from Ambion (MCT1 siRNA: s580 and MCT4 siRNA: s17417), as well as nontargeting control siRNA (Silencer Select Negative Control No.1 siRNA, 4390843, Ambion), using 1 μl/ml of Lipofectamine RNAiMAX (13778-075, Invitrogen), according to the manufacturer's instructions. Since total silencing was only observed after 4 days, being maintained for at least 2 additional days (confirmed by Western Blot), cells were plated at day 3 after silencing and, after overnight adherence, the experiments begin (t0) 4 days after silencing.

### Glucose and lactate measurement

The metabolic behaviour of the cell lines under the different treatment conditions was determined by analysing the extracellular amounts of glucose and lactate. For that, MDA-MB-468 (6,8 × 10^4^ cells/well), MDA-MB-231 (3 × 10^4^ cells/well) and BT20 (4 × 10^4^ cells/well) silenced and control cells were plated in 48-well plates and allowed to adhere overnight. Glucose and lactate quantifications were performed after 24 hours under normoxia or hypoxia conditions. Glucose and lactate were quantified using commercial kits (Roche and SpinReact, respectively), according to the manufacturer's instructions, as described previously [27]. Results are expressed as total μg of three independent experiments.

### Immunofluorescence

MDA-MB-468 (9 × 10^4^ cells/well), MDA-MB-231 (4 × 10^4^ cells/well) and BT20 (6 × 10^4^ cells/well) cells were plated on glass cover slips placed into 12-well plates and allowed to adhere overnight. Cells were then fixed after 24 hours under normoxic and hypoxic conditions. Briefly, cells were fixed with 4% paraformaldehyde during 15 minutes at room temperature and then washed 5 minutes with PBS 1x glicine 10 mM. After, cells were permeabilized with triton 0.1% diluted in PBS 1x for 4 minutes and then washed with PBS 1x (2 × 5 minutes). After blocking with bovine serum albumin 5% (BSA, Sigma Aldrich) for 30 minutes, cells were incubated with the respective primary antibodies: mouse anti-MCT1 (1:200, sc-365501, Santa Cruz Biotechnology), rabbit anti-MCT4 (1:500, sc-50329, Santa Cruz Biotechnology), mouse anti-CD147 (1:200, sc-71038, Santa Cruz Biotechnology) and mouse anti-HIF-1α (1:100, 610958, *BD Biosciences*) diluted in BSA 5%, overnight at room temperature. After washing with PBS 1x (2 × 5 minutes), cells were incubated with fluorochrome-conjugated anti-rabbit (1:500, A11008-AlexaFluor 488, Invitrogen Life Technologies) or anti-mouse (1:250, A11032- AlexaFluor 594, Invitrogen Life Technologies) secondary antibodies, diluted in BSA 5%, for 1 hour at room temperature. Finally, slides were washed with PBS 1x (2 × 5 minutes) and counter-stained with DAPI (Fluoroshield F6057, Sigma-Aldrich).

### Western blot

Cell lysis, protein sample preparation and Western blot were carried out as previously described [27]. Briefly, primary antibodies mouse anti-MCT1 (1:500, sc-365501, Santa Cruz Biotechnology), rabbit anti-MCT4 (1:500, sc-50329, Santa Cruz Biotechnology), mouse anti-CD147 (1:500, sc-71038, Santa Cruz Biotechnology), mouse anti-HIF-1α (1:500, 610958, *BD Biosciences*) and goat anti-actin (1:500, sc-1616, Santa Cruz Biotechnology) were used. Membranes were then incubated with the adequate secondary antibodies coupled to horseradish peroxidase (Santa Cruz Biotechnology) and bound antibodies were visualised by chemiluminescence (Supersignal West Femto kit, Pierce, Rockford, IL, USA). Protein quantification was performed using ImageJ Software (version 1.41).

### Cell biomass analysis

MDA-MB-468 (1 × 10^4^ cells/well), MDA-MB-231 (6 × 10^3^ cells/well) and BT20 (7 × 10^3^ cells/well) silenced and control cells were plated in 96-well plates and allowed to adhere overnight in complete DMEM medium. The effect of MCT knockdown on total biomass, measured by the Sulforhodamine B assay (TOX-6, Sigma-Aldrich), was evaluated after 24 hours of treatment (under normoxia and hypoxia), after a previous 24 hours period of hypoxic growth in the case of the hypoxia treatment condition. Viability curves were calculated with GraphPad Prism 5 software.

### Cell proliferation assay

MDA-MB-468 (1 × 10^4^ cells/well), MDA-MB-231 (6 × 10^3^ cells/well) and BT20 (7 × 10^3^ cells/well) silenced and control cells were plated in 96-well plates and allowed to adhere overnight. Cell proliferation was assessed after 24 hours of normoxia and hypoxia. For the proliferation curves along 10 days, MDA-MB-468 (1000 cells/well), MDA-MB-231 (500 cells/well) and BT20 (500 cells/well) silenced and control cells were plated in 96-well plates and allowed to adhere overnight. Cell proliferation was assessed after 2, 4, 8 and 10 days. To determine the % of proliferation, cells were incubated with 20 μM bromodeoxyuridine (BrdU) for 6 hours before the end of each time point and BrdU incorporation was assessed according to manufacturer's protocol (BrdU, Cell Proliferation ELISA, Roche Diagnostics), as previously described [27].

### Migration assay

MDA-MB-468 (6 × 10^5^ cells/well), MDA-MB-231 (2 × 10^5^ cells/well) and BT20 (3 × 10^5^ cells/well) silenced and control cells were plated in 96-well plates and allowed to adhere overnight. At t0, silenced cell monolayers were washed and a “wound” was made by using a plastic pipette tip. The “wounded” areas were photographed by phase contrast microscopy at 0 and 24 hours. The migration distance was measured using the beWound - Cell Migration Tool (Version 1.5) (developed by A.H.J. Moreira, S. Queirós and J.L. Vilaça, Biomedical Engineering Solutions Research Group, Life and Health Sciences Research Institute- University of Minho; available at http://www.besurg.com/sites/default/files/beWoundApp.zip). beWound is an image processing tool to automate the measurement of cell migration rate in images from Wound-healing assay. This tool comprehends a three step approach: a) automatic image split into cell and “wounded” areas using an approach based on image appearance differences; b) extraction of detailed contours between compacted cell layer and “wounded” area; and, c) measurement of N user-defined lines (5 in current experiments), equally-spaced across the image and perpendicular to the “wound” main axis. This software allows to remove the user-dependency during measurements, while easing the analysis of large databases of images.

The migration distance relative to the control was calculated with the following formula at each time point: relative migration distance (%) = 100 (A–B)/a–b, where A/a is the width of cell wound before incubation, and B/b is the width of cell wound after incubation; A/B refers to the treated condition, a/b refers to the control condition.

### Invasion assay

Cell invasion assay was performed using 24 well BD Biocoat Matrigel Invasion Chambers, according to the manufacturer's instructions (354480, BD Biosciences) and as previously described [27]. MDA-MB-468 (4 × 10^4^ cells/well), MDA-MB-231 (2,5 × 10^4^ cells/well) and BT20 (3 × 10^4^ cells/well) silenced and control cells were plated into invasion chambers for 24 hours either in normoxia or hypoxia. Membranes were photographed in a stereomicroscope and invading cells were counted using the Image J software (version 1.41, NIH). Invasion was calculated as % of cell invasion normalised for the control condition.

### Mouse tumour models

Tumour induction was performed by orthotopic injection of 1 × 10^6^ MDA-MB-468, MDA-MB-231 and BT20 cells silenced for MCTs, in the mammary fat pad of six to eight week-old female N:NIH(s)II: nu/nu nude mice. Tumour growth was monitored weekly using a calliper. At the end of the experiment (45 days), mice were sacrificed by cervical dislocation and the tumours removed, fixed in 10% buffered formalin, embedded in paraffin and sectioned for histological and immunohistochemical evaluations. Tumour volume was calculated according to the formula V = (L × W^2^)/2 (L = length, W = width). Animal experiments were carried out in accordance with the European Guidelines for the Care and Use of Laboratory Animals, Directive 2010/63/UE and the National Regulation published in 2013 (Diário da República, 1.a série- N.o 151-7 de agosto de 2013).

### Histology and immunohistochemistry

A standard Haematoxylin and Eosin (H&E) staining was performed to assess the morphology of tumours. Immunohistochemistry was carried out as previously described [27]. The following primary antibodies were used: mouse anti-MCT1 (1:500, sc-365501, Santa Cruz Biotechnology), rabbit anti-MCT4 (1:500, sc-50329, Santa Cruz Biotechnology), mouse anti-CD147 (1:400, sc-71038, Santa Cruz Biotechnology), as previously described [25], rabbit anti-CAIX (1:2000, ab15086, Abcam) and mouse anti-Ki67 (1:200, AP10243CM, Gennova), during 2 hours at room temperature, using UltraVision Detection System Anti-polyvalent, HRP (Labvision Corporation). Goat anti-PECAM (CD31, 1:400, sc-1506, Santa Cruz Biotechnology) diluted in PBS, was incubated overnight at room temperature, for further incubation with biotinylated horse anti-goat (1:500, BA-9500, Vector Laboratories) secondary antibody, 1 hour at room temperature. In the remaining steps, the R.T.U. Vectastain Elite ABC Kit (Vector Laboratories) was used. Antigen retrieval to Ki67 and CD31 reactions were performed with citrate buffer, in the microwave for 15 minutes.

### Statistical analysis

Data from at least three independent experiments, each one in triplicate, was stored in GraphPad Prism 5 software. All conditions were examined for statistical significance using two-tailed Student's *t*-test for mean comparison, being the threshold for significance *p* values <0.05.

## CONCLUSIONS

In summary, our results show that depletion of MCT1 and MCT4 in breast cancer decrease tumour cell aggressiveness *in vitro* and tumour formation and growth *in vivo*, pointing at MCTs as promising targets for cancer therapy. This work reinforces the previous *in vitro* results [25] and, more importantly, demonstrates for the first time disruption of *in vivo* breast tumour growth by targeting lactate transporters, emphasizing the importance of MCTs in breast tumour initiation and progression.

## SUPPLEMENTARY FIGURES


