# Supplementary material for: Targeting lactate transport suppresses in vivo breast tumour growth
Source: Oncotarget. 2015 May 14;6(22):19177–89. doi: 10.18632/oncotarget.3910 (PMC4662483; doi:10.18632/oncotarget.3910)
Supplement: Supplementary file 1 [file oncotarget-06-19177-s001.pdf]

SUPPLEMENTARY FIGURES

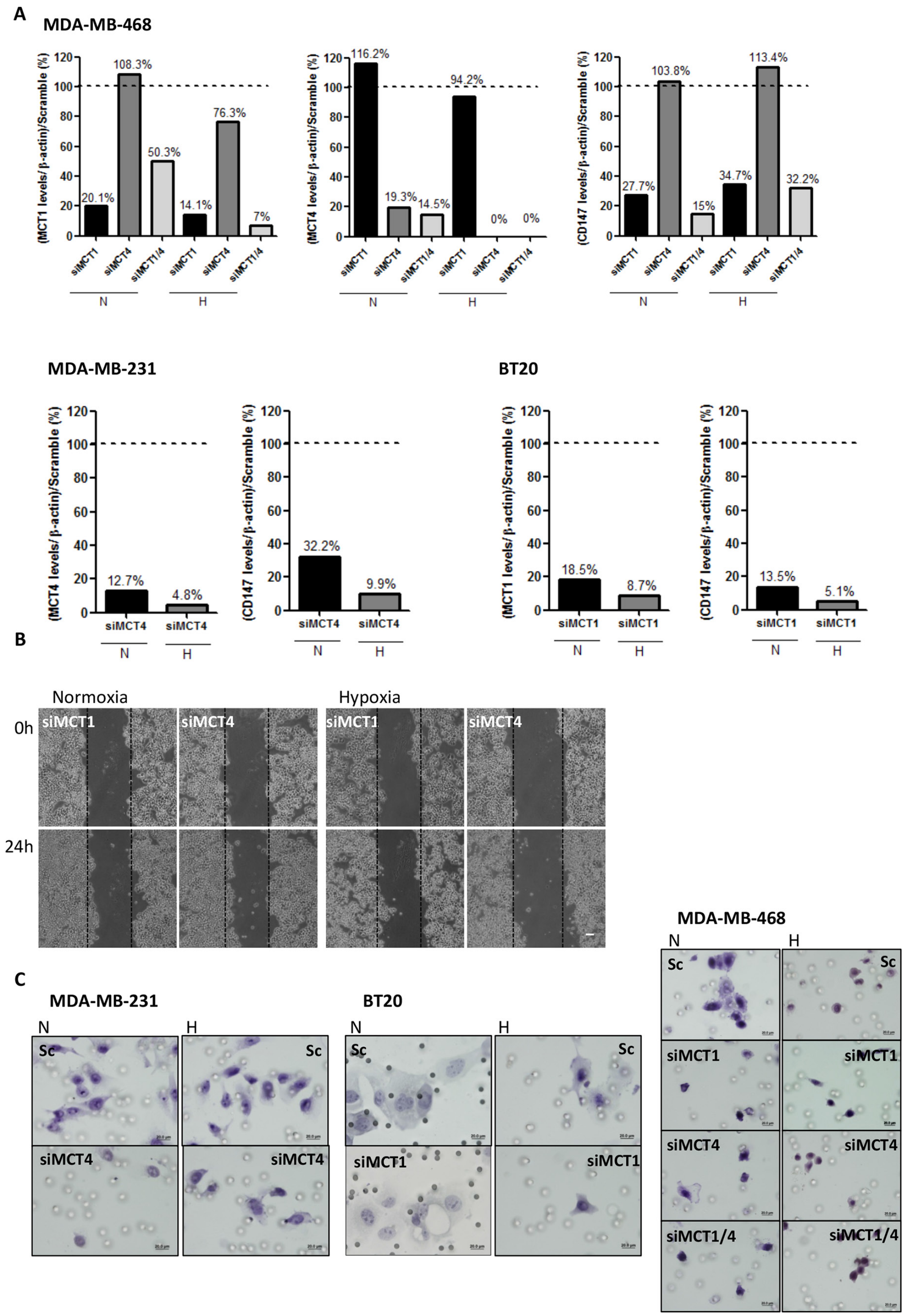

Supplementary Figure S1: Protein levels of MCTs and CD147 and cell motility capacity. A. MCT1, MCT4 and CD147 protein levels after MCT knockdown in normoxia (N) or hypoxia (N), measured after Western Blot analysis, compared to scramble (dashed line-100%). Quantification was performed using ImageJ. **B.** Representative pictures of MDA-MB-468 cell migration at 0 hours and 24 hours after MCT1 and MCT4 knockdown in normoxia or hypoxia (scale bar 100 μm). **C.** Representative pictures of MDA-MB-468, MDA-MB-231 and BT20 cell invasion at 24 hours in normoxia (N) or hypoxia (H) are shown. Silenced cells were compared to the respective control (Sc - scramble). Results are the mean of at least three independent experiments, each in triplicate.

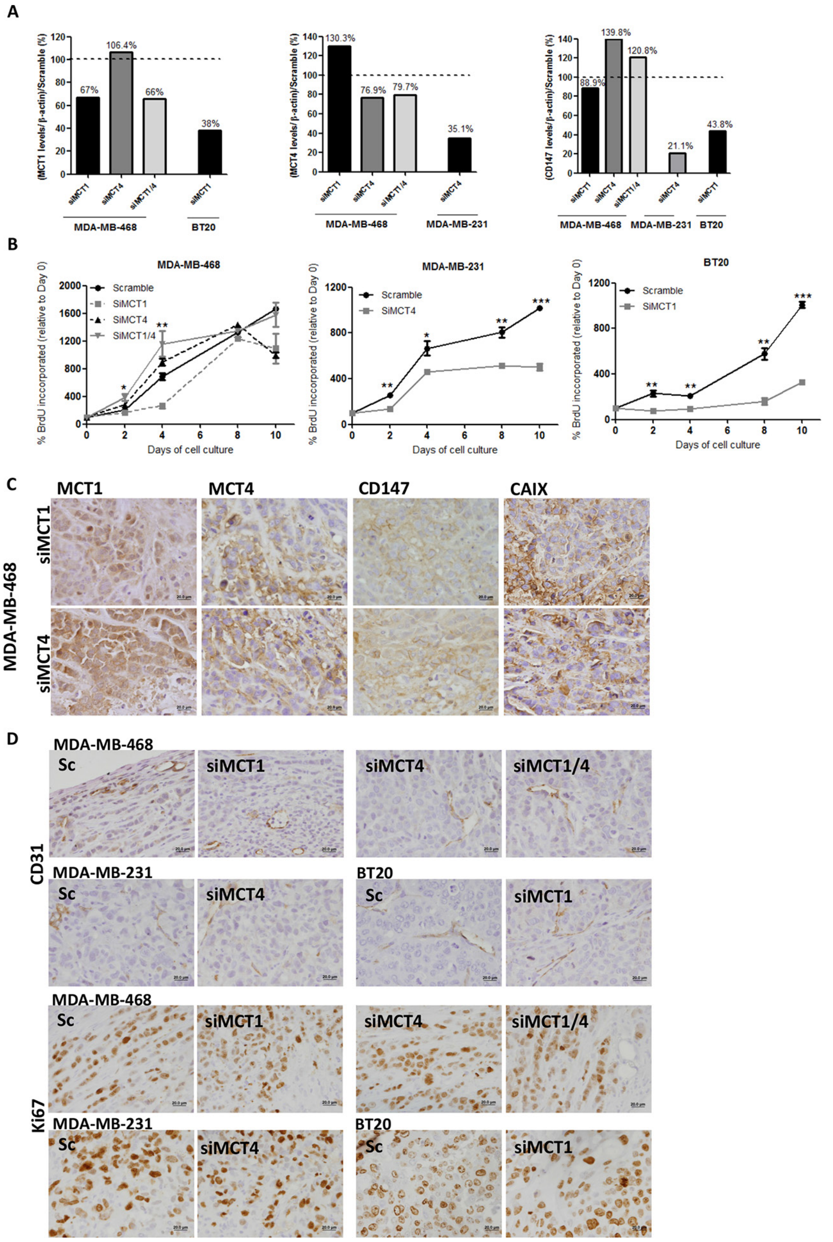

Supplementary Figure S2: Protein levels of MCTs and CD147 after 10 and 14 days of silencing and *in vivo* protein expression. A. MCT1, MCT4 and CD147 protein levels after MCT knockdown in normoxia, after 10 days of silencing in MDA-MB-468 cells or after 14 days of silencing in MDA-MB-231 and BT20 cells, measured after Western Blot analysis, compared to control (dashed line). Quantification was performed using ImageJ. **B.** Proliferation curves of BrdU incorporation along 10 days of cell culture. *: $p < 0.05$; **: $p < 0.01$; ***: $p < 0.001$, siMCT1 (MDA-MB-468 and BT20) or siMCT4 (MDA-MB-231) compared with respective scramble. **C.** Immunohistochemical expression of MCT1, MCT4, CD147 and CAIX in excised MDA-MB-468 tumours after silencing of MCT1 and MCT4 (after 45 Days). **D.** Immunohistochemical expression of CD31 and Ki67 in excised tumours (after 45 Days).
